# Special Issue on Organic Electronic Bio-Devices 

**DOI:** 10.3390/bios3010116

**Published:** 2013-02-28

**Authors:** Luisa Torsi

**Affiliations:** Dipartimento di Chimica, Università degli Studi di Bari, “Aldo Moro”, Via Orabona 4, 70126 Bari, Italy; E-Mail: luisa.torsi@uniba.it; Tel.: +39-080-544-2092

**Keywords:** organic bioelectronics, biomimetic strategies, isothermal amplification methods, paper electronics, barrier tissue, immunoassays, microfibers, conducting polymers

## Abstract

The aim of the present editorial is to briefly summarize the current scientific and technological accomplishments in the field of organic electronic biosensors as described in the articles published in this Special Issue. By definition, a biosensor is a robust analytical device that combines a biological recognition element (e.g., antibodies, enzymes, cells) with a transducer. Organic electronic bio-devices are considered as potentially reliable substitutes of conventional and rather expensive analytical techniques employed for several applications such as medical diagnosis, food safety and environment pollution monitoring. Some insights into the selection and immobilization of recognition elements, signal amplification, fabrication techniques and analytical performance of biosensing devices will be presented.

The emerging field of organic bioelectronics aims at combining in an efficient way biological elements and microelectronic devices for the development of a wide range of portable analytical devices, such as for instance, DNA and protein sensors and Lab-on-a-Chip (LoC) devices. This Special Issue focused on contributions related to bio-electronic sensing devices including organic electrochemical transistors (OECT) integrated with living cells and electrochemical immunosensors based on properly tailored conductive polymers for effective coupling of bio-probes [1,2,3,4,5,6,7]. Further, the dependence of the sensors analytical and electrical performance on parameters such as the nature of functional group, film morphology and size of the immobilized bio-probes has been investigated. Critical aspects related to the design and selection of materials with biomimetic properties and the matching point between molecular recognition and organic electronics in order to improve the sensing performance of the device, are also addressed by the selected manuscripts. Moreover, electrotextile microfibers contain conductive polymers with functional attachment sites for biorecognition elements, as well as the formation of bioactive supramolecular protein layers on inkjet printed electrodes on paper substrates to be used in the development of inexpensive, disposable, point of care electronic devices, have been proposed. The use of isothermal nucleic acid amplification techniques coupled with miniaturized devices for analysis via microfluidics is incorporated in these advancements. 

The field of organic bioelectronics is expanding rapidly worldwide. Recent achievements have led to the development of biological sensing platforms capable of rapid screening of biological samples and point-of-care applications. Organic electronic devices that control cell growth or are used for interfacing with neurons due to their capability to collecting and stimulating electrical signals, are investigated. 

The current Special Issue wishes to share promising strategies of successfully integrated bio-sensing devices and consequently motivate researchers to identify and investigate future application areas in this field.

Several organic bioelectronics devices have been reported in literature, such as electrochemical sensors or organic thin film transistors. In bioelectronic sensors, the biological recognition element is responsible for interacting specifically with a target analyte and the transducer converts the physicochemical event into a digital electronic signal. As transducing elements, modified electrodes with conductive/semiconductive or piezoelectric materials can be employed. Potentiometry, cyclic voltammetry, amperometry and impedance spectroscopy are the most popular transductions principles of bioelectronic sensing platforms. When designing a sensing device the application itself will determine the choice of the structural materials, the fabrication technique and the type of transducer. In general, a biosensor needs to fulfill several requirements such as stability in liquid media, sensitivity, low power consumption, and low cost of manufacture. 

Noble metals in the form of films or nanoparticles, metal oxides, conductive and non-conductive polymers, carbon nanotubes (CNTs) and microfibers are widely employed as materials for the development of functional layers able to bind bio-recognition elements. Different approaches are possible for depositing these materials, such as vacuum evaporation, electro-deposition and even printing techniques. In order to further tailor the aforementioned surfaces to obtain the morphology required for each application, modification methods are explored based on the chemical nature of the materials, the fabrication method, the type of transducer used, the degree of sensitivity and selectivity required, as well as the target biomolecules. For instance in the case of gold or silicon oxide surfaces modification methods involving thiol and silane ligands respectively are used for the formation of self-assembling monolayers (SAMs) or layer by layer assemblies. Moreover a wide range of surface functionalities can be obtained using polymers. Polymers are widely recognized in the literature for their remarkable properties, their biocompatibility, simple synthesis, and easy bio-functionalization. Conductive polymers for example can serve both as being the sensitive components of transducers and as a suitable layer for immobilizing bioreceptors. Selective binding sites can be formed in polymers either by chemical means using different functional groups or by creating template-shaped molds in polymer matrices using molecule imprinting technique (MIP). Additionally, monomers containing biologically relevant side chains or groups available for further functionalization could be electrochemically polymerized to give conductive films displaying such functionality on the surface. Moreover, carbon surfaces are gaining the attention of researches in recent years since they exhibit excellent electrical properties and have a large surface area for interacting with organic species by formation of nanotubes or nanofibers.

In order to sense biocomponents, such as proteins and DNA, or even to achieve high specificity towards a target analyte, biological recognition elements are immobilized on the surface of a sensor. Biological material (e.g., tissue, cell receptors, enzymes, antibodies, aptamers) as well as artificial biomimetic structures (e.g., synthetic receptors, imprinted polymers) are used in order to obtain a surface both selective and sensitive to the target component. 

The integration of biological elements as structural components of an organic electronic device is a crucial step in the preparation of a bioelectronic sensing device. Moreover, application of actual biological species on organic electronic devices requires biocompatibility between the structured materials of the sensor and both the bioactivity of the bioreceptor and electroactivity of the transducing element to be ensured. Tailoring of new biomaterials by bio-genetic engineering allows to create new enzymes, protein receptors, and to generate monoclonal antibodies, aptamers or nucleic acids for non-biological substrates thus allowing easiest integration in electronic devices.

Nevertheless, a key issue in detection of biological species and microorganisms is the limited volume of the analyte sample. Miniaturization of the diagnostic tools in many applications is not always enough to achieve the required detection limits. The use of microfluidics combined with amplification methods of the target analyte is essential in case of DNA detection. Although polymerase chain reaction (PCR) is the most widespread technology for DNA amplification, isothermal amplification processes that offer faster and easier handling can be employed. 

In conclusion, the bio- and surface chemistry related to effective binding of biomolecules on metal/organic surfaces is a relatively new research area but critical to obtain bioelectronics of high performance. The choice of materials to be used as the sensors components is of vital importance and will affect the performance of the sensor. For this reason researchers in this field should determine all essential features that need to be assessed in each specific application.
